# Tinea incognito mimicking pyoderma vegetans

**DOI:** 10.1016/j.jdcr.2026.08.025

**Published:** 2026-09-03

**Authors:** Hesham M. Abdelaziz, Mohamed Khaled, Eman A. Omran, Aya Elbealy, Omar Helal, Omar H. El-Rakhawy

**Affiliations:** a Department of Dermatology, Venereology and Andrology, Damanhour Medical National Institute, General Organization for Teaching Hospitals and Institutes (GOTHI), Egypt; b Department of Dermatology, Venereology and Andrology, Faculty of Medicine, Mansoura University, Egypt; c Department of Microbiology, High Institute of Public Health, Alexandria University, Egypt; d Nancy Hopkins Department of Biology, The American University in Cairo, Egypt; e Department of Odonto-Stomatology, Bellvitge University Hospital, Faculty of Medicine and Health Sciences, University of Barcelona, Barcelona, Spain

**Keywords:** dermatophyte, pyoderma vegetans, tinea incognito, *Trichophyton violaceum*

## Introduction

Tinea incognito is a widely used but etymologically incorrect term; the grammatically correct Latin form is “tinea incognita:” a dermatophyte infection whose clinical features are altered by prior corticosteroid use or other immunosuppression.^1^ “Incognito” lesions often mimic inflammatory dermatoses (eczema, psoriasis, lupus, etc), leading to frequent misdiagnosis and delayed treatment.^2^^,^^3^

Pyoderma vegetans (pyodermatitis–pyostomatitis vegetans) is a rare dermatosis characterized by well-circumscribed exudative, vegetating plaques, usually affecting intertriginous sites (inguinal and axillary folds).^4^

In this report, we describe a diagnostically challenging case of tinea incognito masquerading as pyoderma vegetans in a male patient.

## Case report

A 22-year-old man presented with a 1-year history of an intensely pruritic plaque in the groin. He had been diagnosed elsewhere with pyoderma vegetans after an unrevealing workup (normal blood tests, serum protein electrophoresis, paraneoplastic screening, and colonoscopy). Microbiological evaluation, including fungal and bacterial cultures, was negative.

A skin biopsy from the lesion showed pseudoepitheliomatous hyperplasia with neutrophil-rich intra-epidermal pustules, consistent with a vegetating dermatitis. The patient was given multiple therapies for presumed pyoderma vegetans: oral doxycycline (200 mg/day for 3 weeks), followed by oral prednisone (0.5 mg/kg/day) plus topical steroids and dapsone (150 mg/day). When the lesion failed to improve, cyclosporine (3 mg/kg/day) was added for 3 months, also without response.

On examination, a single 15 × 13 cm moist, verrucous plaque with oozing yellow-brown crusts involved the groin, upper inner thighs, and scrotum (Fig 1, *A*). Hair, nail, and mucous membrane examinations were normal. Because the lesion was persistent and atypical, 3 new punch biopsies were obtained from different areas.

**Fig 1:**
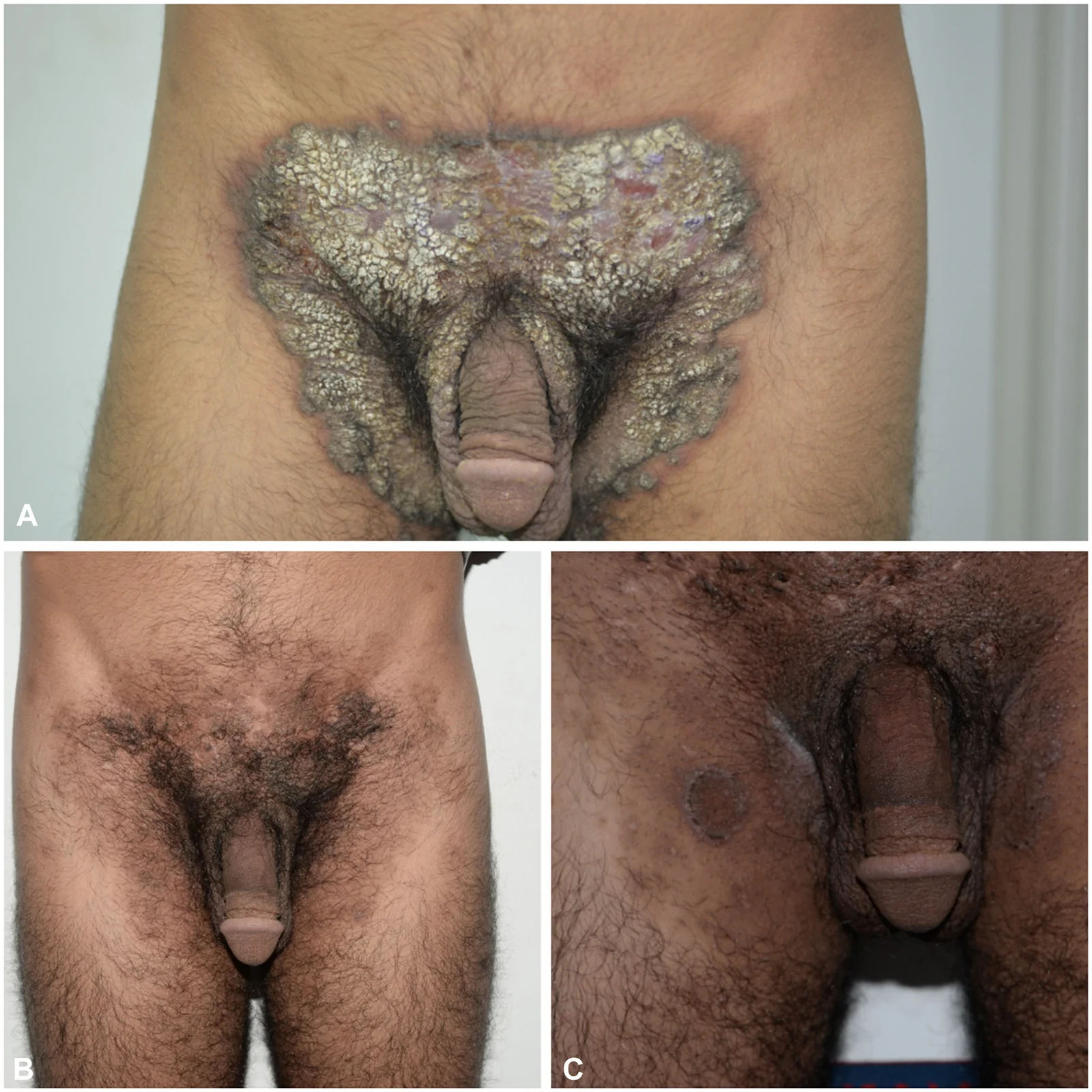
Tinea incognito mimicking pyoderma vegetans. **A,** Large vegetating plaque involving the groin, upper inner thighs, and scrotum before treatment. **B,** Complete resolution after 4 weeks of itraconazole and topical luliconazole. **C,** Recurrence as a well-demarcated circinate, scaly plaque on the inner thigh of the right leg 3 weeks after stopping therapy.

All specimens again showed superficial perivascular lymphocytic infiltrate associated with dilated capillaries at the papillary dermis. The overlying epidermis showed acanthosis, hyperkeratosis, mounds of parakeratosis, and papillomatosis (Fig 2, *A* and *B*). However, fungal hyphae were seen in only one sample: periodic acid–Schiff staining revealed hyphae and spores within the stratum corneum of that specimen (Fig 2, *C* and *D*). Keratolytic debridement of the overlying hyperkeratotic crust was achieved using 4% salicylic acid ointment, and repeat mycological investigations were subsequently performed. Potassium hydroxide preparation and culture of the underlying scrapings yielded *Trichophyton violaceum*. The patient was treated with oral itraconazole (5 mg/kg/day) and topical luliconazole cream 1% twice daily for 4 weeks. The plaque resolved completely (Fig 1, *B*). Three weeks after discontinuation of therapy, a small, well-demarcated circinate, scaly plaque appeared on the inner thigh of the right leg, consistent with recurrent tinea (Fig 1, *C*). A second 3-week course of itraconazole led to complete resolution.

**Fig 2:**
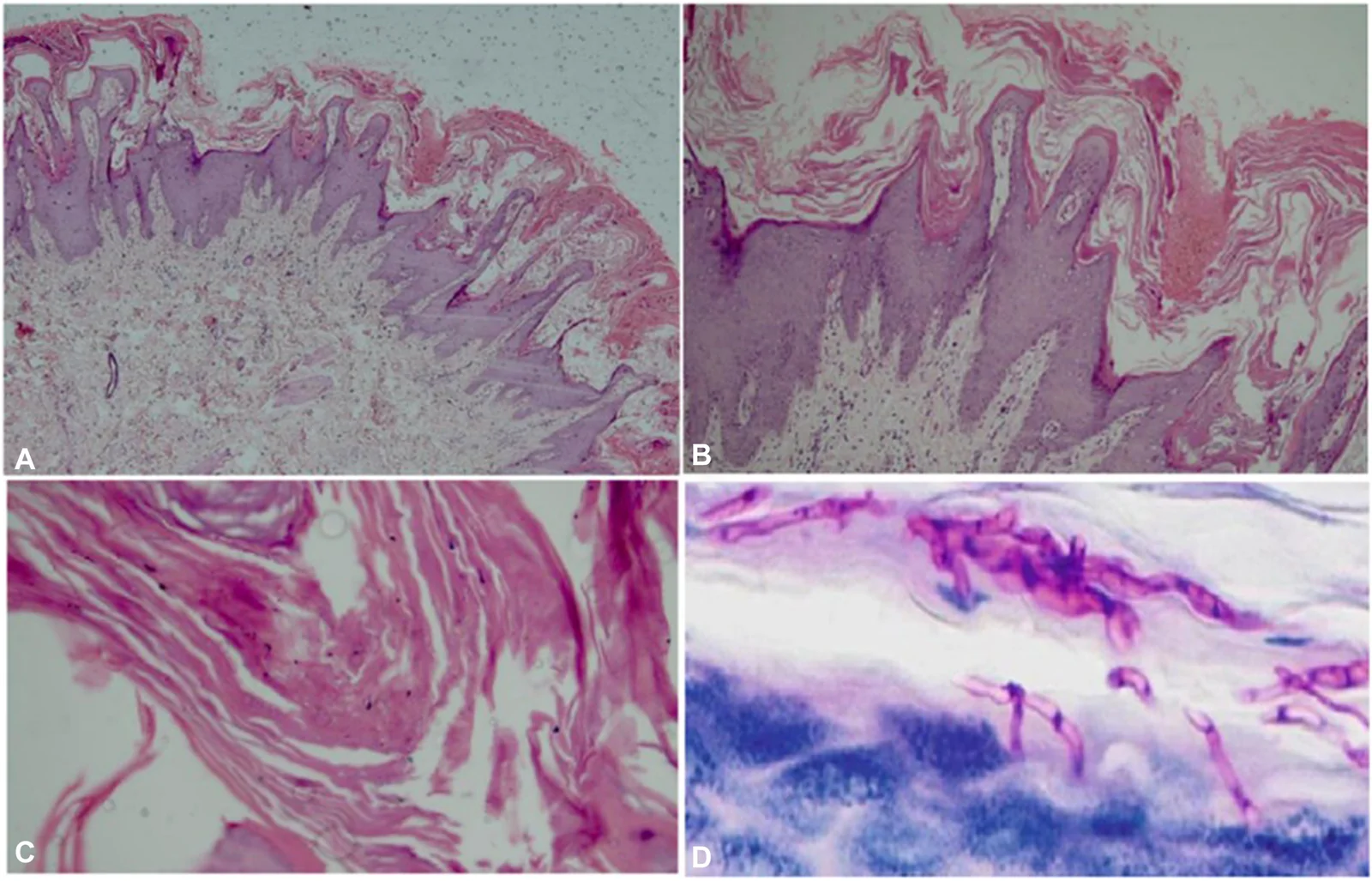
Histopathological findings of tinea incognito. **A,** Acanthosis, hyperkeratosis, and papillomatosis. **B,** Mounds of parakeratosis and intra-epidermal neutrophilic pustules. **C,** Periodic acid–Schiff stain showing fungal hyphae within the stratum corneum. **D,** Higher magnification of periodic acid–Schiff stain demonstrating septate hyphae and spores. (**A** and **B,** Hematoxylin-eosin stain; original magnifications: **A,** ×40; **B,** ×200.) (**C** and **D,** Periodic acid–Schiff stain; original magnifications: **C,** ×200; **D,** ×400).

## Discussion

The initial clinical presentation closely resembled pyoderma vegetans, which is typically associated with eosinophils within the infiltrate and occasionally peripheral eosinophilia, neither of which was observed in this case, leading to diagnostic uncertainty.^5^ However, repeated biopsies with periodic acid–Schiff staining ultimately demonstrated fungal hyphae and spores within the stratum corneum, confirming dermatophytosis.^6^ The initial negative biopsies were likely attributable to an uneven fungal burden and prior corticosteroid or immunosuppressive therapy, both of which may reduce fungal detection. In addition, removal of the thick hyperkeratotic crust, which can impede adequate sampling, was essential for successful fungal culture, ultimately identifying *T violaceum*. These findings underscore the importance of obtaining specimens from beneath hyperkeratotic lesions and performing repeated biopsies with fungal stains when clinical suspicion for tinea incognito remains high despite initially inconclusive investigations.

Although a previous report described pyoderma vegetans developing secondary to recalcitrant purulent dermatophytosis caused by *Trichophyton mentagrophytes*, which was refractory to antifungal therapy and improved only with prolonged antimicrobial and immunomodulatory treatment,^7^ our patient responded rapidly to antifungal therapy alone, and recurrence manifested as typical circinate tinea lesions. This clinical course supports the interpretation that *T violaceum* in the present case clinically masqueraded as pyoderma vegetans (tinea incognito) rather than inducing true pyoderma vegetans.

In the present case, lesions were confined to the groin, pubic region, scrotum, and base of the penis, supporting the diagnosis of tinea genitalis. The patient denied sexual activity preceding lesion onset, making sexual transmission less likely, although this route cannot be definitively excluded on the basis of history alone. Furthermore, no dermatophyte lesions were identified elsewhere on the body, arguing against autoinoculation from another infected site. Mycological examination identified *T violaceum* as the causative organism, in contrast to most reported cases of tinea genitalis, which have been attributed to the *T mentagrophytes*/*Trichophyton interdigitale* complex—including the emerging sexually transmissible genotype VII—or to *Trichophyton rubrum*.^8^^,^^9^ *T violaceum* is an anthropophilic dermatophyte that has become uncommon in many industrialized countries but remains endemic in parts of Africa and the Middle East; its recent re-emergence in certain regions has been associated with migration.^10^ To our knowledge, sexually transmitted genital dermatophytosis caused by *T violaceum* has not previously been reported. Collectively, these findings broaden the recognized clinical, epidemiologic, and mycological spectrum of tinea genitalis.

Tinea incognito is well recognized for mimicking numerous inflammatory dermatoses, including eczema, psoriasis, lupus erythematosus, rosacea, and impetigo.^3^ To our knowledge, this is the first reported case of *T violaceum* tinea incognito presenting as pyoderma vegetans, thereby expanding the clinical spectrum of steroid-modified dermatophytosis. This case highlights the importance of considering dermatophyte infection in atypical, treatment-resistant vegetating lesions, particularly in intertriginous and genital regions. Early mycological investigation, including fungal culture and repeated biopsy with fungal stains, when necessary, may prevent diagnostic delay and avoid inappropriate corticosteroid or immunosuppressive therapy, which can both mask dermatophytosis and promote disease progression and antifungal resistance.

## Conflicts of interest

None disclosed.

## References

[1] Ive F.A., Marks R. (1968). Tinea incognito. Br Med J.

[2] Atzori L., Pau M., Aste N., Aste N. (2012). Dermatophyte infections mimicking other skin diseases: a 154-person case survey of tinea atypica in the district of Cagliari (Italy). Int J Dermatol.

[3] Dhaher S. (2020). Tinea incognito: clinical perspectives of a new imitator. Dermatol Reports.

[4] Adişen E., Tezel F., Gürer M.A. (2009). Pyoderma vegetans: a case for discussion. Acta Derm Venereol.

[5] Mansouri M., Rakhshan A., Shahidi-Dadras M., Karimi A., Alavi S. (2015). Pyoderma vegetans: a case report in a child suspected to primary immunodeficiency and review of the literature. Iran J Med Sci.

[6] Al-Amiri A., Chatrath V., Bhawan J., Stefanato C.M. (2003). The periodic acid-Schiff stain in diagnosing tinea: should it be used routinely in inflammatory skin diseases?. J Cutan Pathol.

[7] Skorepová M., Stuchlík D. (2006). Chronic pyoderma vegetans triggered by *Trichophyton mentagrophytes*. Mycoses.

[8] Jabet A., Dellière S., Seang S. (2023). Sexually transmitted *Trichophyton mentagrophytes* genotype VII infection among men who have sex with men. Emerg Infect Dis.

[9] Kupsch C., Czaika V.-A., Deutsch C., Gräser Y. (2019). *Trichophyton mentagrophytes*—a new genotype of zoophilic dermatophyte causes sexually transmitted infections. J Dtsch Dermatol Ges.

[10] Hoffmann L.C., French L.E., Reinholz M. (2024). *Trichophyton soudanense, Trichophyton violaceum*, and *Trichophyton tonsurans* in Munich: travel- and migration-related cutaneous fungal infections. Dermatology.

